# Online labor trafficking and machine learning: A scoping review and research agenda

**DOI:** 10.1371/journal.pone.0358425

**Published:** 2026-09-24

**Authors:** Rebaka Pradhan, Onn Shehory

**Affiliations:** Department of Information Systems, Bar-Ilan University, Ramat Gan, Israel; Akademia Jagiellońska w Toruniu: Akademia Jagiellonska, CZECHIA

## Abstract

Labor trafficking increasingly intersects with online environments, yet there are limited computational detection approaches. To examine existing methods and their potential applications, this article reviews literature that applies or advocates analytics and machine learning (ML) for detecting labor trafficking (LT). It synthesizes methodologies across two contexts: (a) internet-facilitated LT (IF-LT), where digital platforms enable recruitment and coordination of trafficking, and (b) online-accessible LT (OA-LT), where offline signals of trafficking can be retrieved from online sources. Following Preferred Reporting Items for Systematic Reviews and Meta-Analyses extension for Scoping Reviews (PRISMA-ScR) guidelines, six bibliographic databases were searched between January 2002 and September 2025 and complemented with additional journals, backward and snowball searches, and expert recommendations. Of 2,151 records, we assessed 51 articles for full-text and 21 were mapped and qualitatively synthesized (14 LT-focused and 7 methodologically transferable). IF-LT research clusters around online recruitment, recruitment fraud, illicit massage businesses (IMBs), while OA-LT examines supply chains, child labor, violence indicators on social media, modern slavery disclosures and high-risk sectors such as the fishing industry. Common approaches include case studies with explainable analytics, interpretable classifiers, topic modeling and advanced natural language processing (NLP) methods like sentiment analysis, transformers and large language models; positive-unlabeled learning, probabilistic modeling, and spatial analysis. Despite attention to online LT, the review reveals a gap in quantitative applications of state-of-the-art ML to online content for LT detection. Interdisciplinary and transferable techniques developed for detecting online sex trafficking (ST) offer an adaptable pathway towards automation and law enforcement applications. Beyond methodological gaps, the review emphasizes responsible and context-aware AI, including weighted indicator modeling, remedies for class imbalance, multimodal data integration, label-efficient learning with expert annotations, and clear diagnostic reporting to support accountable deployment.

## Introduction

Labor trafficking (LT) is a worldwide violation of human rights that occurs both domestically and internationally. It involves various actors, such as criminal networks, businesses, employers, governments, consumers, civil society organizations, recruitment agencies, and vulnerable populations. The Protocol to Prevent, Suppress and Punish Trafficking in Persons, supplementing the United Nations Convention against Transnational Organized Crime (hereafter the Palermo Protocol), defines trafficking in persons as the recruitment, transportation, transfer, harboring or receipt of persons, achieved through the threat or use of force, coercion, abduction, fraud, deception, the abuse of power or of a position of vulnerability, or the giving or receiving of payments to obtain the consent of a person having control over another, for the purpose of exploitation [1]. Exploitation is defined to include, at a minimum, the exploitation of the prostitution of others or other forms of sexual exploitation, forced labor or services, slavery or practices similar to slavery, servitude, and the removal of organs. By convention, the trafficking in persons for sexual exploitation is termed sex trafficking (ST) and forced labor and services as labor trafficking (LT). Despite its prevalence, LT remains unevenly visible in research, policy, and technological interventions, particularly when compared with ST. This imbalance reflects not only differences in empirical accessibility, but also deeper asymmetries in how exploitation is conceptualized, operationalized, and detected, especially in digital environments.

**Fig 1:**
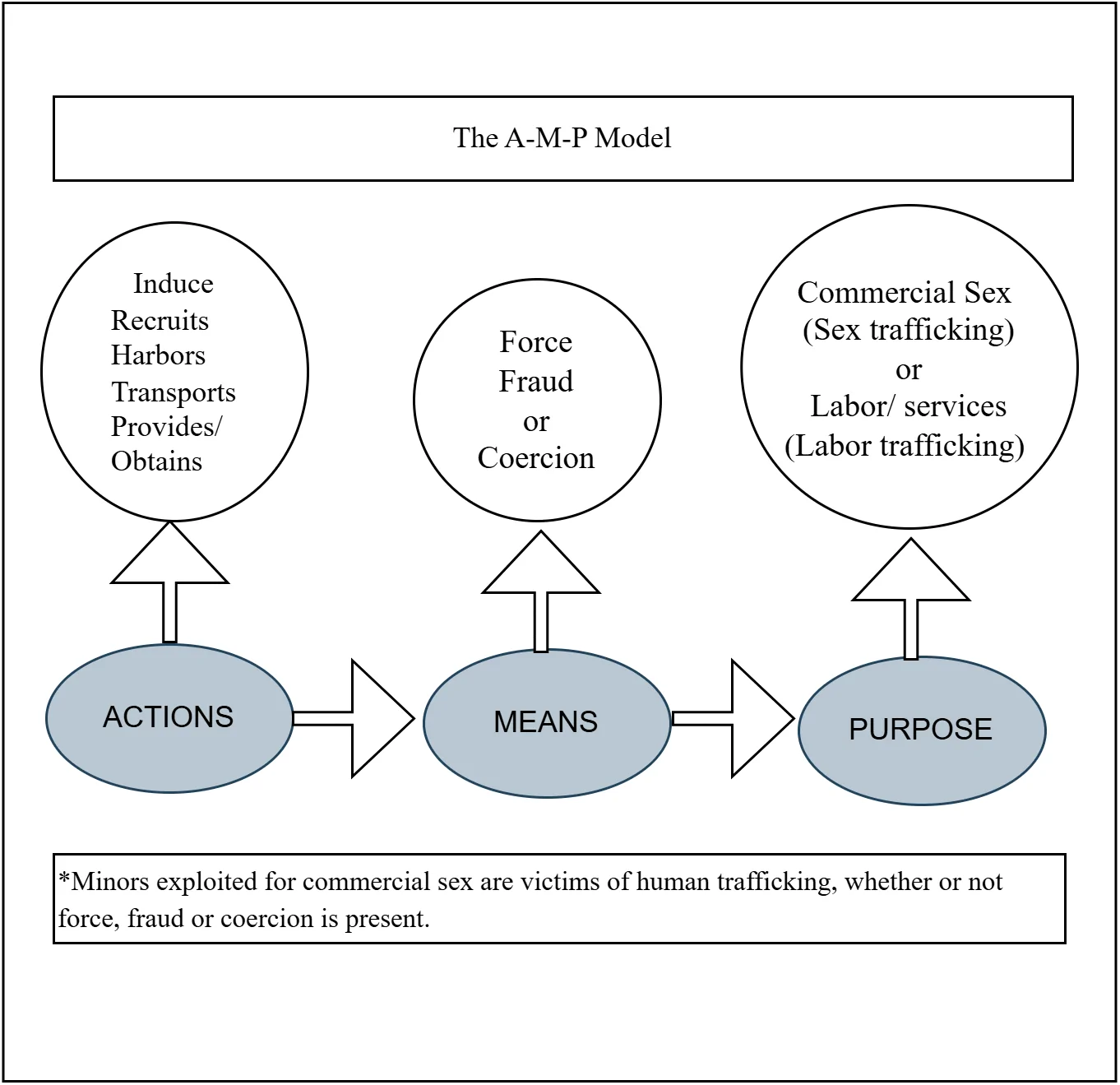
The Action-Means-Purpose (AMP) model of identifying victims of trafficking. Produced by the authors based on the AMP model described by Polaris [4].

This phenomenon is primarily observed in the unskilled labor sector, which is subject to less regulatory scrutiny and often experiences seasonal fluctuations in labor demand [2,3]. The Action-Means-Purpose (AMP) model illustrates the trafficking process and the route taken by a trafficker; at the minimum, the presence of one element from each column (Fig 1) points to a potential trafficking instance [4].

Recognizing the prevalence of LT, the United Nations (UN), International Labour Organization (ILO) and various institutions have produced comprehensive indicator lists of human trafficking (HT). Volodko et al. [5] operationalized these indicators for the identification of trafficking signals in job advertisements targeted at migrant job-seekers. The Global Report published by the UN emphasized the growing use of internet-based platforms for illicit activities, particularly in the context of recruitment, noting that almost half of the victims were affected by online recruitment among the set of court cases analyzed [6].

In the field of analytics and operations research, there is a significant focus on ST, despite LT being estimated to account for more than 60% of all trafficking instances. However, LT only constitutes 12% of the studies in a prior review [7]. This disparity is particularly salient in the application of artificial intelligence (AI) and machine learning (ML). One of the prominent computational applications addressing forced labor is satellite detection of brick kilns [8], whereas the methodological maturity, labeled datasets, and benchmark tasks are far more developed for ST than for LT [9,10].

Research that employs ML techniques specifically for online LT detection is even more limited [11,12]. This highlights the disproportionate representation of LT compared to ST, as indicators of sexual exploitation in online contexts are more explicit, while LT indicators are typically indirect, ambiguous and context dependent. Also, the broader HT literature often equates HT with ST, rather than distinguishing between trafficking types and their distinct exploitative purposes [13–16]. As a result, AI-based approaches risk inheriting and amplifying these conceptual biases. This, in turn, shapes which forms of exploitation become computationally legible and which remain underrepresented.

In response to this gap, this review consolidates existing efforts that use analytics and ML to detect LT across two contexts: internet-facilitated LT (IF-LT), where recruitment or coordination is done via digital platforms such as online recruitment, recruitment fraud, and illicit massage businesses (IMBs); and online-accessible LT (OA-LT), where relevant signals can be retrieved online although operations occur offline, such as in supply chains, child labor, violence indicators on social media, modern slavery disclosures, and high-risk sectors such as the fishing industry.

Importantly, the application of AI in these contexts is not merely a technical exercise, but a socio-technical intervention with potential consequences for workers, employers, and enforcement institutions. Misclassification, surveillance, and uneven data availability raise questions of accountability, transparency, and appropriate human oversight – issues that are central to contemporary debates on AI and society.

While prior reviews have examined human trafficking broadly, often focusing on sex trafficking, there is still no consolidated synthesis of machine learning applications specifically addressing labor trafficking across both internet-facilitated and online-accessible contexts. As a result, existing work remains fragmented, making it difficult to compare methods and identify common challenges in applying machine learning to labor trafficking detection.

To address this gap, this review maps and synthesizes existing methodologies and the context in which they are applied, and examines their implications for future AI-assisted labor trafficking detection. The review is guided by the following research questions:

1. Mapping: What is the state of labor trafficking research that applies or advocates the use of machine learning or analytics across IF-LT and OA-LT contexts?
2. Synthesis: What can be learned from the existing scientific evidence and countermeasures to improve online labor trafficking detection through responsible and context-aware AI methods?

## Methods

In developing the review method, we were guided by the Arksey and O’Malley [17] and Joanna Briggs Institute (JBI) guidance [18], which include (1) identifying the research question, (2) identifying relevant literature, (3) screening relevant studies based on the inclusion criteria, (4) data charting, and (5) discussion of results. The reporting is guided by the Preferred Reporting Items for Systematic Reviews and Meta‑Analyses extension for Scoping Reviews (PRISMA-ScR) [19] ensuring documentation and reproducibility.

Prior to the review process, a protocol to guide the search, eligibility criteria and data-charting procedures was developed by the authors’ team to ensure methodological transparency because scoping reviews are not eligible for PROSPERO [20] registration. The Protocol summary is available in S2 File. In order to identify relevant literature we created a search and selection strategy which is described below:

### Search strategy

We used the JBI Population-Concept-Context (PCC) framework (Table A in S1 File) to consolidate and operationalize the strategy for the search. The population details the trafficking phenomenon related to labor trafficking, modern slavery, and trafficking networks. The concept of interest is the analytical and machine learning techniques applied to trafficking detection or analysis of trafficking-related activities. The context encompasses the digital or online-accessible environments in which trafficking signals can be observed.

The search was systematically carried out for literature published between January 2002 and September 2025 with boolean, block-based search strings to ensure replicability (Table B in S1 File) across six databases namely SpringerLink, IEEE Xplore, Taylor & Francis, ACM Digital Library, ScienceDirect and Scopus. The detailed database search strategies and retrieved records are available in Table C in S1 File. The starting date (2002) was chosen to capture two decades of development in this field following the adoption of the Palermo protocol, which catalyzed attention and reporting on trafficking, leading to subsequent research. These databases contain the majority of the relevant literature, providing international and interdisciplinary coverage, where studies related to human trafficking are typically published. Secondly, we conducted manual searches across ArXiv and ResearchGate for conference articles to capture emerging literature prior to their formal publication. Third, we ran backward searches on reviews and snowball searches for mapped articles. We also included ‘labour trafficking’ in addition to ‘labor trafficking’ as specific industries used the term interchangeably in various academic and grey areas like webpages, policy proceedings and media etc. Search terms were selected to broadly reflect the HT literature, informed by the first author’s domain expertise, and finalized collaboratively by the author team.

### Eligibility criteria

To achieve the aims of this review, we focused on the articles addressing LT in online contexts. The study includes the literature that apply or explicitly advocate ML or analytics, rather than discussing LT conceptually or theoretically. Online LT in this review refers to both IF-LT and OA-LT data that can be derived from the internet such as recruitment boards, job reviews, web content, open geodata or remotely sensed. The included studies had to be in English, published as peer-reviewed journal articles or conference papers, preprints, or doctoral dissertations. Grey literature in the included set comprises the preprints and dissertations. Additional grey literature, namely agency and institutional publications, supports definitional, contextual and indicator-framework claims and was not screened against these criteria. As the LT-specific ML literature is sparse, we also conducted targeted searches of online ST literature to extract transferable methodologies (as demonstrated, e.g., by [21], and cited by ILO [22]). These studies are also included in the data charting but reported in a separate column as methodological articles. Studies related to IMBs, which are commonly known for their association to sexually oriented services and may operate as a hybrid of LT and ST, were included under the LT category because of the involvement of these environments in deception and coercion in the recruitment and exploitation of victims. This is based on the Polaris findings from trafficking hotline massage business cases, in which survivors reported some form of labor trafficking in all cases.

### Study selection

Records retrieved from database searches were imported into Rayyan systematic review software [23] for a two-stage screening (S2 File). This includes title and abstract screening followed by full-text eligibility assessment. Studies were evaluated against the inclusion criteria. Screening decisions were discussed between the authors for agreement on inclusion.

### Data charting

A standardized data charting form (Table 1) was produced to extract key study characteristics such as research type, the data source, country represented in the study, techniques and methods, key findings and a separate column showing the category whether the articles directly refer to LT or methodological transferability.

**Table 1:** Mapping of reviewed studies.

#N	Author (year)	Title	Research type	Data source	Country	Techniques and methods	Key findings	LT	MT
1	Volodko et al., 2020	Spotting the signs of trafficking recruitment online: Exploring the characteristics of advertisements targeted at migrant job-seekers	A case study- suggesting automating with machine learning	Job advertisements from a Lithuanian website.	Lithuania	Content analysis of job advertisements.	Identifying indicators of human trafficking within the advertisements.	Yes	
2	Cascavilla et al., 2022	Unsupervised labor intelligence systems: A detection approach and its evaluation	unsupervised machine learning approach	Facebook groups and pages focused on job advertisements.	Netherlands	Topic modeling (Latent Dirichlet Allocation and Latent Semantic Indexing) and logistic regression.	Logistic regression for prediction of salary discrepancies	Yes	
3	Tobey et al., 2024	Interpretable models for the automated detection of human trafficking in illicit massage businesses	Machine learning modeling	Yelp customer reviews, U.S. Census data, and GIS data.	United States (specifically Texas and Florida)	Interpretable machine learning models, including risk scores and optimal decision trees.	Prediction modeling of businesses involved in trafficking	Yes	
4	White et al., 2024	Why are you here? Modeling illicit massage business location characteristics with machine learning	Machine learning for spatial analysis	Online advertisement data scraped from websites like RubMaps, U.S. Census data, and other public datasets.	United States	Random Forest models for classification and inferential modeling.	Identifying locations of IMBs	Yes	
5	Thöni et al., 2018	An information system for assessing the likelihood of child labor in supplier locations leveraging bayesian networks and text mining	Applied research using a combination of machine learning and probabilistic modeling	Publicly available news articles, social media data, and statistics on child labor.	Global scope, but the study is particularly relevant to regions with higher child labor risk.	Bayesian Networks for probabilistic modeling, text mining to extract relevant incidents from online data.	Bayesian networks compute the likelihood of child labor at supplier locations with dynamic updates from online data sources.	Yes	
6	Nallakaruppan et al., 2023	Child tracking and prediction of violence on children in social media using natural language processing and machine learning	Machine learning and natural language processing (NLP)	Social media data from Twitter, collected using APIs and web scraping tools.	Global	VADER sentiment analysis, Decision Tree algorithm, and the APIFY web scraper for data collection.	The model classifies sentiment to detect potential child trafficking on social media.	Yes	
7	Joo et al., 2023	Towards a responsible machine learning approach to identify forced labor in fisheries	Applied research using machine learning for practical application.	Vessel characteristics, movement patterns, Automatic Identification System (AIS) data.	Global	Positive-unlabeled learning with machine learning classifiers, Random Forest classifiers.	The model predicts the likelihood of forced labor use based on movement patterns and vessel characteristics.	Yes	
8	Li et al., 2023	Detecting human trafficking: Automated classification of online customer reviews of massage businesses	Applied machine learning	Customer reviews from Yelp and Rubmaps.com, two different types of platforms with varying levels of explicit content.	US – 5 metropolitan cities	NLP, deep learning, Ensemble learning	Pattern detection, optimization of measurements like precision and recall	Yes	
9	Ouyang R, 2023 (Thesis)	Machine learning for tangible effects: Natural language processing for uncovering the illicit massage industry & computer vision for tactile sensing	Applied machine learning	Publicly accessible websites like Google Places, Rubmaps, and AMP Reviews forum. This includes reviews, business locations, and business attributes. Also, synthetic transaction data	US- across 50 states	NLP, agent-based modeling for synthetic datasets, transformer models	Pattern detection of illicit business. Using business hours as one feature for classification.	Yes	
10	Nersessian and Pachamanova, 2022	Human trafficking in the global supply chain: using machine learning to understand corporate disclosures under the UK modern slavery act*	Machine learning for textual analysis.	Corporate disclosures (17,000 MSA statements) from companies- web	UK	Natural language processing (NLP), topic modeling.	Analysis of themes and compliance patterns in corporate disclosures to assess engagement with human trafficking risks.	Yes	
11	Tobey, 2023 (Thesis)	Data science and machine learning methodologies for detecting human trafficking risk in the illicit massage industry	Prediction modeling	Data from Yelp and Rubmaps.com reviews US census data	US	NLP, ML, AL	Predict high risk businesses	Yes	
12	Garg et al., 2025	Detecting illicit massage businesses by leveraging graph machine learning	Applied machine learning / graph analytics	IMB related advertisements	US	Graph machine learning, network analysis, feature engineering	Graph ML improves identification of trafficking-linked IMBs	Yes	
13	Zhou et al., 2024	Tracing the unseen: Uncovering human trafficking patterns in job listings	Applied Machine Learning	Chinese-speaking online platforms in the United States 2006–2024 (over 250,000 job postings)	US	Textual analysis, clustering, temporal analysis	suspicious job ads, often linked to human trafficking, increase during crises		Yes
14	da Silva Santos et al., 2019	Machine learning models to identify the risk of modern slavery in Brazilian cities	Applied research using machine learning models	Socioeconomic and demographic data, rescue operations data.	Brazil.	Logistic Regression (LR) and Gradient Boosting Machine (GBM), with Random Over-Sampling (ROS) for handling imbalanced datasets.	Predictive modeling		Yes
15	Alvari et al., 2017	Semi-supervised learning for detecting human trafficking	Computational Predictive Modeling	Online classified advertisements, backpage.com	Global scope, with specific focus on U.S.	Semi-Supervised Learning, textual analysis, Graph based analysis	effectiveness of semi-supervised learning with a regularization term over baseline supervised and unsupervised methods		Yes
16	de Vries and Radford, 2022	Identifying online risk markers of hard-to-observe crimes through semi-inductive triangulation: The case of human trafficking in the United States	qualitative interviews and computational text analysis.	Online reviews of illicit massage businesses and expert interviews	US	Word embeddings (a NLP technique)	The study identifies online risk markers for human trafficking in IMBs		Yes
17	Rodriguez Perez and Rivas, 2023	Combatting human trafficking in the cyberspace: A natural language processing-based methodology to analyze the language in online advertisements	Applied machine learning	Online escort ads -Skip the Games (STG) website	US	NLP, Transformer models for NER	Generating pseudo labeled datasets with minimal supervision		Yes
18	Mendez Guzman et al., 2022	RaFoLa: a rationale-annotated corpus for detecting indicators of forced labor	Text classification	News Articles	Global	Annotation, Transformer models, multi- label and multi- classification, NLP	rationale-annotated corpus focused on forced labor to support multiclass and multi label classification validated on transformer based baseline models.	Yes	
19	Bora et al., 2025	AIMSCheck: leveraging LLMs for AI-assisted review of modern slavery statements across jurisdictions	Text classification	Modern slavery statements- 50 Australian statements	Australia, UK, Canada	NLP, LLMs	Fine tuning of LLMs performed better over zero and few shot models in assessing compliance in MSA's	Yes	
20	Hanif et al., 2024	Machine learning approach in predicting fraudulent job advertisement	Predictive modeling for Text classification	Employment Scam Aegean Dataset (EMSCAD)- 17880 job ads	US, Britain, Canada	Logistic Regression, Support Vector Machine, Decision Tree, and Naïve Bayes algorithms combined vectorizers.	Classical ML models can be combined with text vectorizations in identifying fraudulent ads that can be extended to job ads too.		Yes
21	Kejriwal et al., 2017	FlagIt: A system for minimally supervised human trafficking indicator mining	Applied, system building – Minimally supervised machine learning	Online escort ads from DARPA	US	Unsupervised text embeddings and semi supervised heuristic re-labeling	Indicator mining system in limited label settings		Yes

Note. LT: studies directly addressing labor trafficking or labor exploitation. MT (methodological transfer): studies from sex trafficking or related domains whose methods, features, or datasets are transferable to LT detection. Each study is marked in one column only, reflecting its primary contribution to this review.

### Quality appraisal

In accordance with JBI guidance for scoping reviews, no formal quality appraisal of included studies was conducted. The objective of the review was to map and synthesize existing methodological approaches rather than evaluating study effectiveness.

## Results

### Study selection process

**Fig 2:**
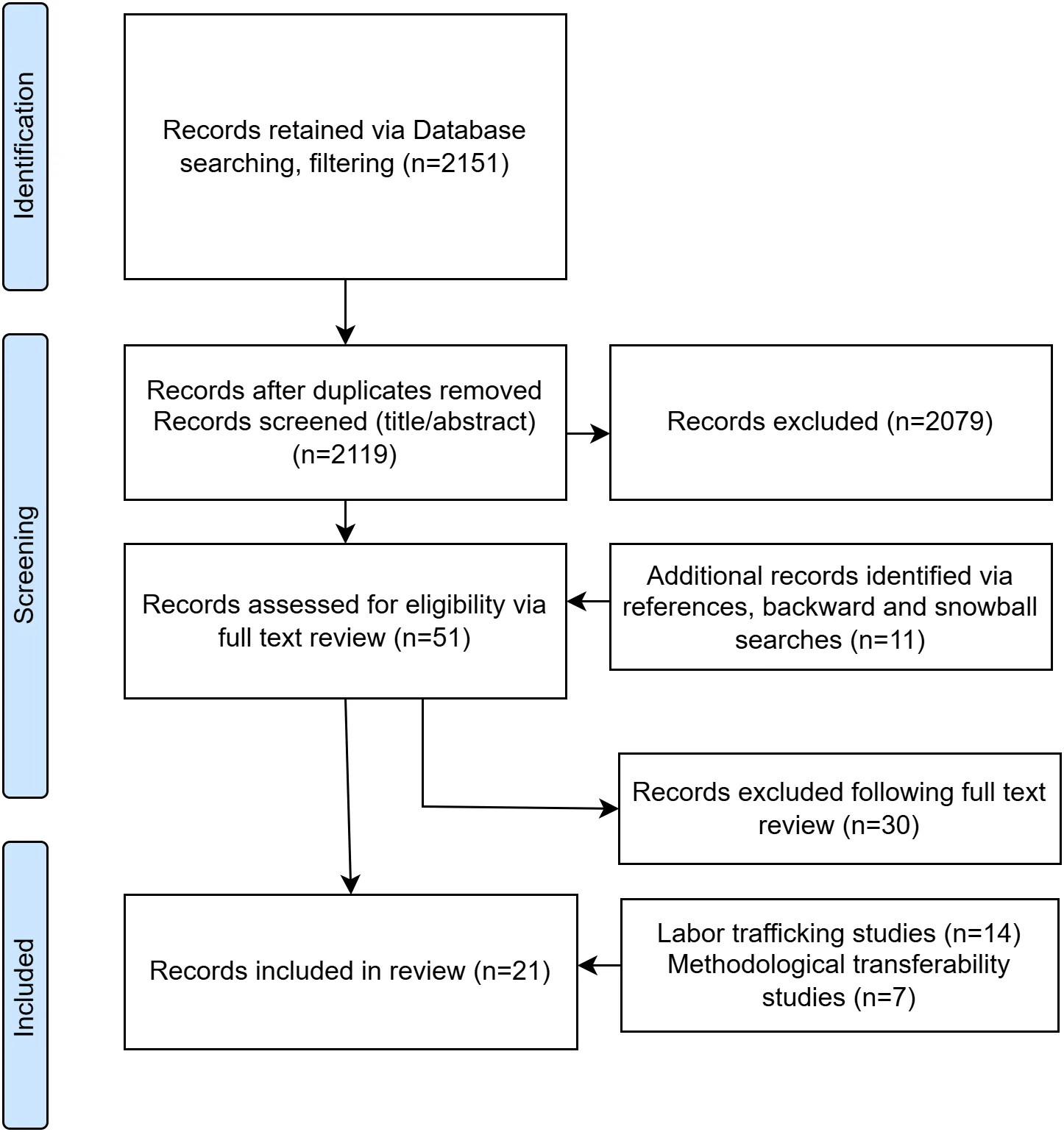
PRISMA flow diagram summarizing study identification, screening, eligibility assessment, and inclusion.

The study selection process is illustrated in the PRISMA flowchart (Fig 2). The full database yields were imported into the Rayyan systematic review software tool. Those that could not be imported were separately managed in Excel with standard bibliographic fields such as authors, title, year, source, abstract, digital object identifier. Following the database-specific search and export-stage filtering, 2,151 records were retained for deduplication and screening. After deduplication using the title string and author names, 2,119 unique articles remained and went through title and abstract screening. While transitioning to full-text assessment, we also identified 11 articles via snowball sampling, citation tracking, and expert recommendations, including some important references. In total, 51 studies were retained and proceeded to full-text eligibility check based on the inclusion criteria. At the full-text eligibility stage, studies were excluded if they were unrelated to labor trafficking contexts, did not involve data sources, did not apply analytics or machine learning or did not suggest a data-driven way forward. Because the LT-based data-driven literature is limited, seven additional studies were included for their methodological transferability (MT), taken from sex trafficking contexts which could inform labor trafficking detection. After full-text screening, we conducted final agreement on eligibility and methodological adequacy. This resulted in the final set of 21 articles: 14 LT-focused and 7 MT studies.

### Mapping of included studies

Table 1 presents the mapping list of the 21 included studies as a data extraction table that records the article name and the characteristics such as the research type, the data source, country represented in the study, techniques and methods, key findings and a separate column showing the category if the articles directly refer to LT or methodological transferability.

Literature addressing IF-LT and OA-LT is concentrated in a few geographies, namely Lithuania, the Netherlands, the United States, the United Kingdom, and Brazil. Most datasets are country-specific and based on labor information. Studies draw on diverse online sources, including web-based recruitment data for unskilled labor, social media data such as Facebook and Twitter, Geographic Information System (GIS) data, business reviews, publicly accessible websites (e.g., Google Places), online news, official labor statistics, synthetic data, corporate disclosures, case articles, and interviews published online. There are also analyses of data scraped from websites that flagged trafficking risk. The majority of research employing automation techniques was conducted between 2018 and 2025, suggesting a growing interest in automation-based analytical approaches. This trend underscores both the growing need and the expanding scope for scalable methods, as well as the increasing relevance of research addressing the online dimensions of LT. Much of this research concentrates on online recruitment, particularly in job advertisements and forced labor in industries such as fisheries. A significant focus is placed on the illicit massage industry which is a hybrid form of LT alongside ST. Literature also mentions child labor and exploitation in supplier locations. The categorization of methodologies for detecting LT includes content analysis and exploratory studies, as well as predictive modeling, classification tasks and spatial analysis. Traditional ML techniques such as Naive Bayes, Logistic Regression, Support Vector Machines (SVM), and Random Forest models with decision trees are commonly used for classification, clustering and inferential modeling, with logistic regression also being combined with topic modeling in certain cases. Probabilistic approaches like Bayesian Networks and positive-unlabeled (PU) learning with ML classifiers are utilized for handling uncertainty. Natural Language Processing (NLP) and text analysis play a key role, employing advanced techniques like transformer models BERT, CNN, and LSTM, along with general deep learning models and large language models (LLMs). Sentiment analysis (e.g., VADER) further supports the detection of exploitation.

Overall, the evidence base is limited, with only a handful of analytical and ML intervention based studies. Qualitative designs in LT detection research such as field studies, policy analyses and interpretative studies outweighed quantitative ones. Limited data accessibility, with ST often treated as synonymous with HT, often obscures the studies that capture specific aspects of LT.

## Discussion

This section first clarifies the relations between the concepts: forced labor, trafficking and slavery. It then discusses the result set theme-wise and concludes the review’s implication into future research.

### Conceptual distinction in concepts related to labor trafficking

**Fig 3:**
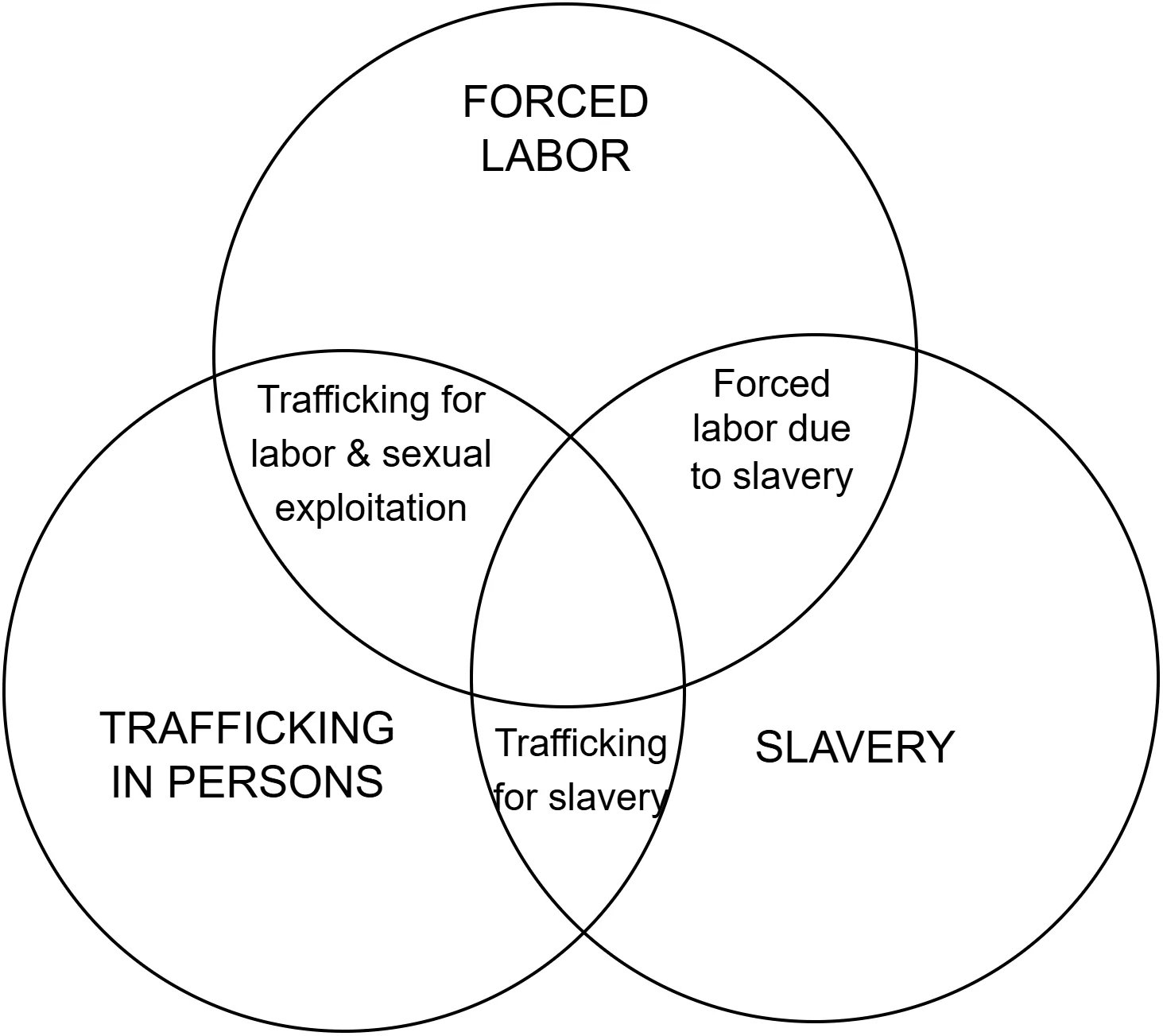
Conceptual overlap between forced labor, human trafficking and slavery. (Produced by the authors based on the definitions from ILO, “What is forced labour?”).

Researchers argue that LT is best understood as part of a continuum of exploitation lying between decent labor and forced labor [24–26] causing harm to workers and the reputation of industries. The literature reviewed in this article mentions the terms forced labor, human trafficking (also called “trafficking in persons”), slavery and modern slavery. As set out in the Introduction, HT constitutes a single offense under the Palermo Protocol, within which ST and LT are distinguished by the purpose element rather than by act or means [1]. Forced labor is work exacted under the menace of any penalty and not offered voluntarily, the two elements being cumulative [27]. Slavery denotes the status or condition of a person over whom powers attaching to the right of ownership are exercised. Modern slavery, by contrast, is an umbrella term used in advocacy and vary across jurisdictions whose scope has been seen in the literature [25,26]. These concepts overlap substantially in practice (Fig 3). Trafficking as a mechanism, with progression, can lead to forced labor and, in the extreme, can escalate to a state of slavery. Forced labor and slavery are defined by the condition or status of the person, whereas trafficking is defined by the process leading into that condition. Hybrid cases, of which IMBs are the clearest instance, involve labor exploitation and sexual exploitation of the same workforce and therefore satisfy more than one enumerated purpose simultaneously.

### Methodological implications

**Fig 4:**
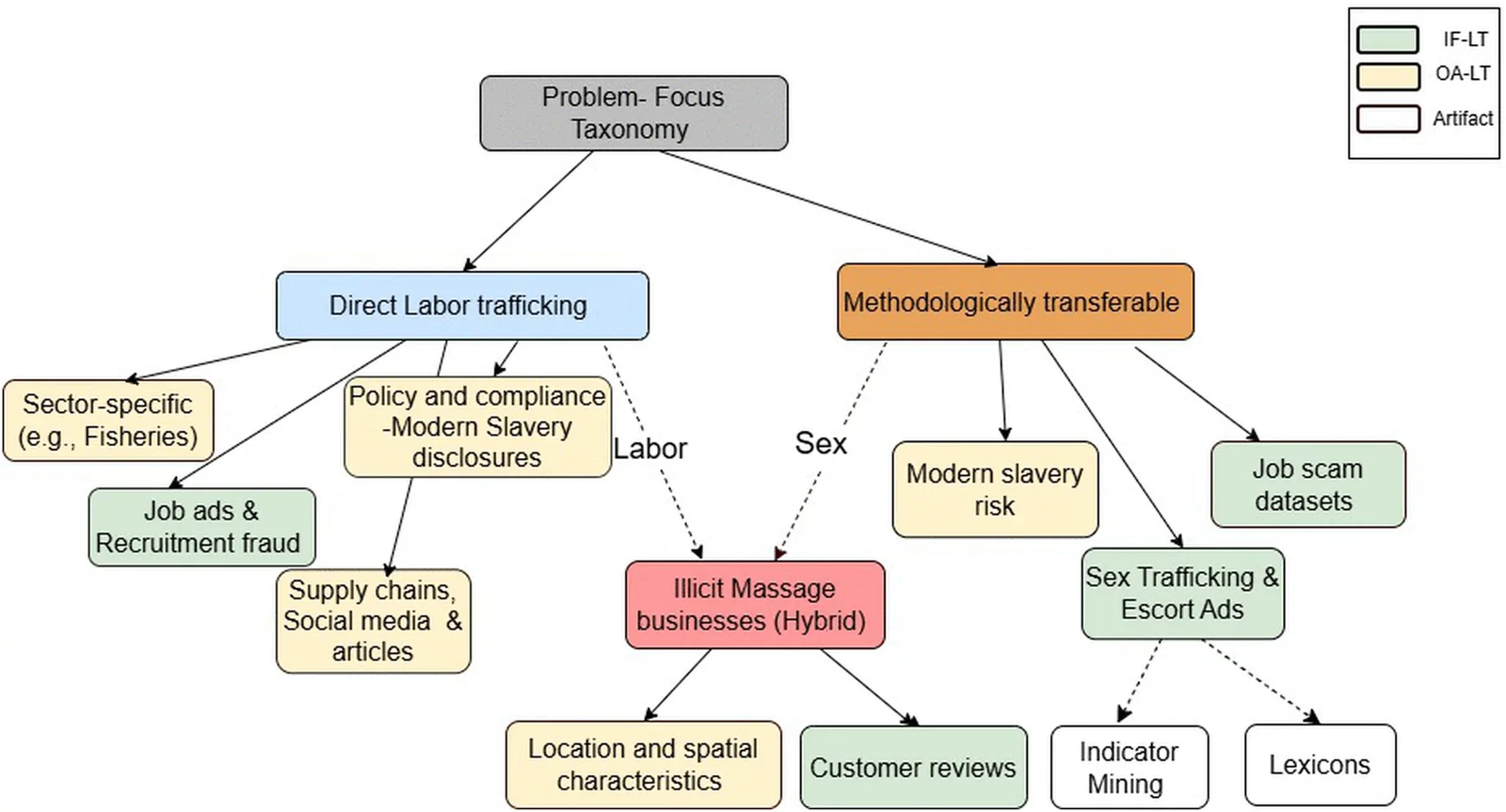
Problem-focus taxonomy of labor trafficking detection.

The themes across the literature pertaining to LT applying or advocating ML techniques include job advertisements for labor recruitment leading to trafficking and job advertisements for scam, IMBs, modern slavery risks in supply chains, child labor, violence on social media, Modern Slavery Act disclosure analysis and articles annotation; and one of the high-risk sectors such as the fishing industry alongside methodologically transferable insights from escort ads in ST detection literature. These themes and their subproblems are represented in the taxonomy diagram (Fig 4).

The taxonomy situates the data sources and their analytical contexts across the reviewed studies for LT, the methodologically transferable ST domain, and the hybrid IMBs context. Apart from the data charting that maps the literature reviewed here, the discussion offers a synthesis chart of transferability and design proposition (Table 2) for developing systems to detect online LT complementing the narrative that follows.

**Table 2:** Synthesis of theme and methodological implications.

Theme/Domain	Key features identified in studies (#)	Transferability & design for online LT detection
Labor job ads (Recruitment)	Wages, vague descriptions, operational indicators, demographic targeting, combinational indicators (Refer: #1, #2, #13, #20)	1. Combine weak and strong indicators; weight features on predictive strength; active learning for borderline ads 2. Integrate wage/hour information with other contextual indicators to flag suspicious cases 3. Use metadata, linguistic deception cues and network analysis to trace recruiters 4. Address class imbalance through RUS, SMOTE, cost-sensitive learning or hybrid approaches
Illicit massage businesses (Hybrid)	Reviews, sentiment, location, business attributes (Refer: #3, #4, #8, #9, #11, #12, #16)	1. Combine interpretable models with ensembles and additional data (reviews, financial, contract terms, recruiter metadata) 2. Develop multimodal algorithms (spatial, demographic, economic (e.g., recruitment hubs, points of entry/exit, high-risk sectors)) with textual data; 2-stage models to handle zero-inflated data 3. Balance precision/ recall by combining lexicons and embeddings, expand with domain-specific vocabulary 4. Combine expert interviews with text analysis to capture implicit cues, extend with seed based semantic risk networks. 5. Model relationships between the advertisement and business entities.
Supply chains / modern slavery disclosures	Child labor risk, compliance, rationale-annotated corpora (Refer: #10, #19)	1. Apply topic modeling with supervised classifiers 2. Use sentence-level classification with token-level explainability; leverage prediction probabilities for reviewer confidence
Social media / online platforms- reports & articles	Social media sentiment, rationale annotations (Refer: #5, #6, #14, #18)	1. Integrate diverse information (case reports, news, interviews) to enrich detection models. 2. Use feature selection (LASSO) and imbalance-handling techniques (ROS, SMOTE, ADASYN); incorporate urban–rural distinctions in risk assessment. 3. Apply rationale-based annotation (e.g., ILO, TA Hub, BHRRC) for multi-label/multi-class classification; extend to job ads interpretability
Labor-intensive sectors (Fisheries)	Vessel movement, socio-demographics (Refer: #7)	1. Target deceptive ads in fisheries. 2. Apply Positive-Unlabeled (PU) learning for scarce positive cases.
Escort ads (Recruitment)	Entity recognition, pseudo-labels, contact networks (Refer: #15, #17, #21)	1. Apply regularization on unlabeled data to improve performance 2. Generate pseudo-labels for interpretable predictions, apply transformer models 3. Combine weak supervision with adaptive learning; retrain with positive label probabilities; apply efficient text embeddings (e.g., FastText ‘bag of tricks’)

Note. Numbers in parentheses refer to the study numbers (#N) in Table 1.

With globalization [28] and the large-scale proliferation of affordable internet facilities, the transnational HT market has expanded beyond borders, within the illicit and licit sectors [13]. In terms of the AMP model (Fig 1), exploitation commonly uses deception at recruitment [3], such as promising wages higher than the market norms [29]. Recognizing this, across the recruitment domain, considerable research starting with an early foundational work [9], shaped global policies and technological interventions in this domain. The literature suggests that online job ads contain actionable signals such as violations of the national wage-and-hour standards, vague job role descriptions, off platform redirects, benefits such as accommodation, transport to work, transfer to destination country and help with settling although not necessarily indicate trafficking but can be present to mask the exploitative conditions [5,30]. Upon analysis it was observed that indicators usually co-occur rather than being present in isolation and only 10 out of 59 indicators laid out by the UNODC [31] could be operationalized, highlighting the challenges of using indicators developed for offline use in the online sphere. Community-specific platforms such as for the Chinese-speaking immigrants to the US refer to cultural and linguistic patterns in job advertisements. This is essential in mitigating bias in online HT detection [32] making the model more interpretable with preemptive measures [33]. Related work on recruitment fraud [34] shows overlapping tactics such as mass recruitment, offer of attractive packages, and typical writing style. These works suggest exploring topic modeling to extract thematic information and leveraging LLMs for analysis and reporting, as traditional ML models require extensive training data, LLMs may be helpful to outperform them in classification tasks [35]. While these studies demonstrate the potential of ML for recruitment-stage labor trafficking detection, they are largely limited to single platforms, specific linguistic, cultural context, and indicators characterizing a particular set of industries [5,30,33,34]. The most persisting gap is the absence of ground truth validation against confirmed cases and limited reporting of evaluation metrics. At the dataset level, some advertisements with too short texts were removed for reliable topic extraction [30], potentially biasing the dataset and results. In addition, heavily imbalanced datasets in this domain require resampling techniques while mitigating the bias.

IMBs exemplify a distinct hybrid combination and facilitation of both LT and ST [36]. These businesses advertise massage or therapeutic services disguised as legitimate businesses, but exploit workers through low wages and unlawful working hours, confiscating passports, sometimes coupling these services with sexual exploitation. Interpretable models with ensemble methods and features enriched with review text, licensing status, business attributes such as opening hours, recruiter metadata and geo-demographic indicators can surface risk while preserving transparency for decision-makers [37,38]. Spatial correlates such as proximity to airports and ports, neighborhood type, and local cost structures helped forecast relocation after raids and shutdowns [39]. Active learning pipelines for labeling NLP tasks in the absence of labeled data and alternative sources such as the Google Places API extend coverage [40,41]. In parallel, triangulation frameworks from computational criminology show how linguistic markers derived from embeddings, context-aware lexicon and gender guesser can be combined with geospatial layers to produce more robust, multi-dimensional risk assessments [42]. More recently, graph based ML also has been explored to model relationships across the complex information in advertisements and business entities to improve detection [43]. The IMB studies are predominantly US-based and trained on data from specific states such as Texas or Florida limiting both geographic generalizability and reproducibility of findings. The interpretability of models specifically with risk scores and decision trees trades off the accuracy because they use simpler models in order to achieve the transparency. Such a methodological gap can be addressed with explainable AI techniques.

The recruitment into massage industries for labor exploitation that occurs through deceptive online job advertisements, places IMBs within the internet-facilitated category (IF-LT) [36]. However, the detection methodologies applied by researchers rely predominantly on post-hoc online-accessible traces such as customer reviews, business location data and operating hours [37–41,43], which are characteristic of OA-LT detection contexts. The IF-LT and OA-LT categories therefore capture two distinct analytical aspects. One, through which trafficking is facilitated and the other, digital traces available for detection. This explains how to design the computational detection in either case. Detection systems targeting IMBs may therefore benefit from integrating both recruitment-stage signals alongside post-hoc consumer signals for a broader detection approach.

Within the supply chain contexts, important multi-source monitoring of information comes from social media, news, articles, audit data on child labor and other broader modern slavery risks. These are based on the demand-side evidence rather than recruitment or source-side detection which should be more preventive [44,45]. In this scenario, the UK Modern Slavery Act is a groundbreaking legislative approach to combating HT within corporate supply chains by requiring companies to disclose their anti-trafficking efforts with its “reporting mandate”. Analysis of these statements emphasize high-level policies rather than just the operational measures to detection [46,47]. Although modern slavery monitors the disclosure, it cannot verify the actual compliance that limits their utility in mitigating exploitation. Predictive modeling with lasso regularization on highly imbalanced data from socioeconomic, demographic, and rescue operations to identify modern slavery risk in Brazil can be further extended to multiclass models in urban and rural settings. Such data can be integrated to the information accessed online which are highly imbalanced to draw better inference on risk.

Recent work provides evidence for the applicability of LLMs to assist human reviewers with the system providing sentence level decisions, token level rationales and calibrated probabilities in assessing model’s confidence in its predictions. The AIMSCheck system [35], shows how LLMs can move beyond the binary classification towards rational clarity, guiding human reviewers through lengthy modern slavery compliance documents, and supports more informed decision-making. It is also seen that Fine-tuned models outperformed zero-shot and few-shot approaches in the compliance classification task [35,48], suggesting that models should be context-aware for reliable performance. Together, these developments imply accountable AI deployment in labor trafficking mitigation.

Often the lack of ground truth constrains fully supervised systems. To address this, RaFoLa [49] offers a rationale-annotated corpus for detecting indicators of forced labor drawn from news articles from traffick analysis hub, the Business and Human Rights resource center, and ILO newsroom for annotation which is rationale-oriented and derived mainly from the indicators list from the ILO. While this corpus reflects the demand-side evidence which is the public reporting of the incident, rather than the preventive recruitment-stage detection, it can be extended by developing sentence-level justification, improving label efficiency and supporting multi-label classification of co-occurring indicators in the online recruitment context.

Sector-specific analysis of a prominent hotspot of forced labor and trafficking is on the fishing industry which notes that vessels involved with forced labor exhibit systematically different behavior from other vessels. Combined together with vessel telemetry, technical characteristics, PU signs of forced labor could be predicted [50]. While this analysis does not include economic, social, or political causes of forced labor, data integration with other labor dynamics such as point of entry of worker, recruitment characteristics through recruitment agencies both online or offline recruitment, living conditions of workers, point of exit for the worker along with the restrictions imposed in leaving the workplace such as debt bondage or unemployment [51] would allow a comprehensive assessment of forced labor at sea.

Additionally, methodologies from ST domain, such as the semi-supervised model conducted by [21] offers adaptability for LT detection as recommended by ILO [22]. It is based on language patterns, words-of-interest, and victim characteristics. Similarly [52], develops a methodology for interpreting language in customer-to-customer (C2C) marketplace ads with pseudo-labels requiring minimal human intervention for training and evaluating NLP models. Lightweight, efficient text embeddings such as the “bag-of-tricks” [53] approach with heuristic relabeling have also shown advantages for large-scale screening compared with heavier paragraph embedding baselines in similar domains [54] and can also be translated for LT scenarios. It is a good example of a system that can operate in limited label settings by careful combination with proven ML techniques. Collectively, these studies suggest a practical direction as outlined in the way forward section.

Across the studies, methodological robustness varies considerably. The recruitment-stage studies present foundational but exploratory work with relatively smaller datasets and evaluation reporting. These studies showcase a methodological progression from indicator counting [5] to unsupervised ML [30] to computational analysis [37] and supervised classification [34]. Trafficking detection studies across IMBs are more developed with applied [37,40,41], interpretable [38] ML, spatial analysis [39], qualitative triangulation [42] and graph ML [43]. These report quantitative metrics across larger datasets containing reviews and advertisements. For example, studies on IMBs employ structured review datasets combined with census and geospatial data. This enables the reporting of precision–recall trade-offs and risk scores, in contrast to recruitment-stage studies where evaluation is often limited or absent. Supply chain studies [44] mostly rely on demand-side evidence such as rescue operation data [46], proprietary modern slavery statements [47], with LLM approaches as the most advanced design [35]. The fishing industry study addresses a distinct high-risk sector but remains constrained by label scarcity [50]. As an example, the fisheries study applies positive-unlabeled learning to vessel telemetry data to address the label scarcity. This is distinct from IMBs or supply chain studies where proxy labels from customer reviews and corporate disclosures provide a structure for model training. The geographic and methodological coverage across these studies are diverse but limits cross-country comparison due to the different application areas. Methodologically transferable studies [21,52,54] offer adaptable approaches, though validation on labor-specific datasets is needed. Together these patterns point to the need for standardized evaluation frameworks and shared benchmark datasets to advance methodological maturity in this field.

### Structural constraints

The methodological limitations that constrain the field's maturity are best understood not as incidental gaps that a better model would resolve, but as structural constraints around data, methodological fragmentation, and context-bound transferability, which reinforce one another. The most fundamental is the data problem, which is the absence of a stable computational target. Because trafficking is hidden and legally contested, researchers rarely have confirmed cases and instead rely on proxies, but are not ground truth instances itself, so a model's reported accuracy reflects agreement with the proxy rather than detection of trafficking itself. Constitutive elements of offense such as coercion, debt bondage and document retention are realized inside the employment relationship, after recruitment, so recruitment-stage data cannot contain them, and deception requires the advertisement to appear lawful. The weak discriminative signal is therefore a property of the offense rather than a deficiency of the data. This also partially restricts transfer of trained signal from sex trafficking, where the advertisement is the point of sale and the artifact analyzed carries the transaction itself; in labor recruitment it describes conditions that do not yet exist, which is why the strongest available signals are economic irregularities within lawful documents rather than evidence of an offense. Fragmentation follows whichever records an institution happens to generate. Because these records are created for different purposes, they encode different phenomena, whether consumer perceptions, corporate compliance reports, enforcement outcomes, or behavioral anomalies. Consequently, datasets capture different constructs rather than different observations of the same phenomenon. Even when studies operate within the same domain, their findings cannot be readily synthesized because they measure fundamentally different aspects of exploitation. Hence, successive studies cannot readily build on one another. Together, these structural constraints determine what a detection system can and cannot see, and in turn who is scrutinized and where intervention is directed.

### Socio-technical implications and governance considerations

The application of AI and machine learning to online LT detection should be understood not just as a technical enhancement of investigative capacity, but as a socio-technical intervention that reshapes how exploitation is identified and acted upon [55]. Given the ambiguous nature of the online trafficking indicators, models risk identifying proxies for exploitation rather than the actual instances. Algorithmic systems inevitably reflect the assumptions embedded in their data sources, labels, and modeling choices, thus influencing which forms of labor exploitation become visible to institutions and which remain obscured.

A central implication concerns computational visibility. As this review indicates AI-based approaches to LT rely heavily on indirect and context-aware indicators, often derived from fragmented or unevenly available online data. For example, studies focusing on IMBs [37–39] rely only on the customer reviews and make exploitation visible only when digital traces exist. These constraints risk privileging certain regions, sectors (e.g., construction and domestic work), or forms of exploitation that are more observable online. Consequently, AI systems may reinforce existing blind spots in LT research and enforcement, rather than correct them.

Another implication relates to risk and accountability [56]. Misclassification in LT detection has non-trivial consequences: false positives may stigmatize legitimate businesses or workers, while false negatives may leave exploitative practices unaddressed. Unlike many commercial AI applications, errors in this domain intersect directly with human rights and legal processes. These stakes emphasize the need for transparency in model design, clear reporting of uncertainty, and mechanisms for review by human decision-makers.

The reviewed literature also raises concerns regarding surveillance and power asymmetries. Many AI-driven approaches depend on large-scale monitoring of online content, supply-chain disclosures, or social media activity including vessel telemetry [50], corporate disclosure analysis [47], and social media scraping [45]. While such data may be publicly accessible, their aggregation and algorithmic analysis can expose already vulnerable populations such as migrant or informal workers, while affording limited visibility into the practices of more powerful actors. This asymmetry highlights the importance of carefully delimiting the scope of automated monitoring and embedding AI tools within governance frameworks that protect individual rights.

Finally, these findings point to the necessity of human-in-the-loop and institutional safeguards. AI systems for LT detection should be designed to support, rather than replace, expert judgment by investigators, regulators, and civil society organizations. Incorporation of domain expertise during model development, validation, and deployment can help mitigate bias, improve contextual sensitivity, and enhance trust. Moreover, decisions about which tasks to automate and which to leave to human discretion, should be informed by legal, ethical, and organizational considerations, not solely by technical feasibility. AIMSCheck [35] exemplifies this through sentence-level justifications without replacing human judgment.

Together, these socio-technical considerations suggest that progress in AI-enabled LT detection depends as much on governance, accountability, and institutional design as on advances in data and algorithms. Responsible deployment requires viewing AI not as an autonomous solution, but as a component within a broader ecosystem of social, legal, and policy interventions. As a scoping review, this study does not evaluate or benchmark the empirical performance of individual models, but instead synthesizes methodological approaches, data sources, and conceptual assumptions across the existing literature.

### Way forward

Building on the theme-based synthesis in the preceding section, this review identifies methodologies and the transferability for advancing research that can detect LT at scale. Importantly, the objective of such systems is not to ascertain individual cases of LT, but to flag suspicious instances for closer human examination. As the discussion notes, indicators play an important role in shaping detection systems; they should be modeled not as simple binary tags but as a combination of weak and strong indicators. This can improve discriminatory power by weighting features on predictive strength rather than treating them as binary.

Beyond the existing literature, future work should focus on the integration of heterogeneous data and the systematic combination of automation with expert input. One possible approach is the use of active learning and expert labeling of borderline ads coupled with feature importance. This can refine indicator weights efficiently while penalizing noisy indicators, and can be further supported by weak supervision frameworks to auto-generate probabilistic labels in label-scarce settings. Wage and hour information, which is an empirically strong indicator, should be integrated with contextual features to generalize the ability of a model. Lexicons are valuable for precision and embeddings for recall, combining them in ensembles can balance performance when expanded with domain-specific vocabulary to strengthen coverage. Likewise, unstructured corpora benefit from topic discovery so that the extracted features can be used alongside the supervised classifiers for potential exploitation detection.

As the majority of ads are non-exploitative, two-stage models are appropriate to handle zero-inflated data distributions. In scenarios where features are numerous relative to confirmed positive cases, regularization and class imbalance should be treated with a combination of data-level methods (random under-sampling and over-sampling) and synthetic variants such as Synthetic Minority Over-sampling Technique (SMOTE) and Adaptive Synthetic Sampling (ADASYN) and algorithm-level approaches (e.g., cost-sensitive loss functions).

While detection tools can aid law enforcement, their deployment may also introduce risks, such as the over targeting of migrant workers or excessive surveillance of vulnerable populations. Such tools and systems should serve as a decision-support rather than autonomous detection. Accordingly, the evaluation of such tools should extend beyond technical performance metrics to include potential harms and unintended social consequences for affected populations [57]. Taken together, the mapping and synthesis of the literature highlights the need to integrate additional data sources, develop adaptable lexicons, operationalize the knowledge for decision making, reinforce active learning, and empirically test the indicators alongside reporting practices. In particular, transparent reporting of precision, recall, specificity, sensitivity and calibration together with false-positive consequences is essential to support responsible application in real-world settings [55].

Moving forward, our forthcoming research aims to translate some of these approaches into a unified, context-aware pipeline that bridges data, methodology and detection systems for online LT detection, while maintaining explicit attention to accountability, interpretability, and human oversight in deployment contexts.

### Limitations

This scoping review is subject to limitations and sources of potential selection biases across its scope, evidence-base and source-composition. Firstly, the search process was restricted to English-language publications, potentially excluding relevant studies in other languages with significant data-driven research in non-English speaking regions. Secondly, despite searching six databases, the review may have omitted some relevant qualitative or conceptual studies across grey literature, and reports addressing labor trafficking through other methodological approaches. Thirdly, consistent with scoping review guidelines, this review does not perform a formal quality appraisal or comparative evaluation of model performance across the identified data sources.

Additionally, across the studies there is a heterogeneity in reporting of the study application success. While some provide evaluation metrics others provide qualitative findings, making relative comparison of effectiveness difficult across methods. Accordingly, the synthesis is exploratory in nature, mapping the methodologies in the existing research rather than ranking them. Finally, the relatively limited volume of data-driven research in this domain also influenced the representation and scope of the reviewed literature. For example, the scope of the regions mentioned in the included studies particularly United States, United Kingdom, Lithuania, Netherlands, Australia, Canada, Britain and Brazil with dominance of US-based studies, shows a geographic skew limiting the data representations and hence context-based applicability of findings across geographies and contexts.

Beyond the scope, a constraint in this field of study is the accessibility of datasets across the included studies, as they considerably vary in terms of accessibility. Some studies draw on fully publicly accessible sources such as Yelp reviews [37], job advertisements [5,30], open government data and news articles [49], where independent replication is possible. Some datasets are owned by organizations, such as the compiled Modern Slavery Act statement databases [47], and may be partially accessible upon request from the authors. Others are restricted to law enforcement or government agencies, including rescue operations data [46], and cannot be accessed by independent researchers. Studies examining IMBs scraped data from RubMaps platform have migrated domains following law enforcement pressure [37–41,43] effectively inaccessible for replication from the same source. A similar constraint applies to methodologically transferable studies where platforms such as Backpage.com have been shut down [21] and certain datasets remain law enforcement restricted [54]. This inaccessibility constrains reproducibility of the studies and limits improvement of the models. This can be mitigated by the creation of ethically curated, anonymized data accessible upon request to researchers.

Finally, two features of the evidence base introduce considerations for interpretation of the findings. The inclusion of grey literature such as preprints and dissertations broadens coverage and offers valuable methodological insight, but this work has not undergone peer review, so its findings are more indicative than confirmatory. Similarly, the sex-trafficking studies were included in the discussion for their methodological transferability rather than their subject matter; even though they inform a useful range of tested methods, the conclusions that rest on them remain conditional on the transferability limits discussed earlier. Both choices reflect the sparse LT-specific literature and were necessary for adequate coverage to further the research in this domain. They widen the evidence base at some cost to homogeneity and should be weighed accordingly.

## Supporting information

S1 File PCC Framework, Search query, database specific search strategy, query details. (DOCX)

S2 File Review protocol summary. (DOCX)

S1 Checklist PRISMA-ScR-Checklist. (PDF)
