# Supplementary material for: Online labor trafficking and machine learning: A scoping review and research agenda
Source: PLoS One. 2026 Sep 24;21(9):e0358425. doi: 10.1371/journal.pone.0358425 (PMC13606961; doi:10.1371/journal.pone.0358425)
Supplement: Supplementary file 2 [file pone.0358425.s001.docx]

# **Supporting Information**

**S1 File. PCC Framework, Search query, database specific search strategy, query details**

**Table A. P-C-C Framework tabulating population, concept, and context in trafficking research**

| **Population** | **Concept** | **Context** |
| --- | --- | --- |
| labor trafficking | prediction | Dark web |
| modern slavery | machine learning | social media |
| trafficking | text mining | Supply chain |
| forced labor | natural language processing | Dark net |
| forced labour | Social network analysis | virtual markets |
| child labour | data mining | marketplace |
| trafficking networks | knowledge management | classifieds |
| human trafficking | deep multimodal models | Review sites |
| labour trafficking | active learning | cyber* |
| child labor | sentiment analysis | advertisements |
|  | indicator mining | social networking sites |
|  | statistical analysis | labor markets |
|  | network analysis |  |

**Table B. Search terms**

| **Shorter search string** | (“labor trafficking” OR “forced labour” OR “trafficking networks”)  AND  (“dark web” OR “social media” OR “advertisements” OR “labor markets”)  AND  (“machine learning” OR “social network analysis”) |
| --- | --- |
| **Comprehensive search string** | (“labor trafficking” OR “modern slavery” OR “trafficking” OR “forced labor” OR “forced labour” OR “child labour” OR “trafficking networks” OR “human trafficking” OR “labour trafficking” OR “child labor”)  AND  (“dark web” OR “social media” OR “supply chain” OR “dark net” OR “virtual markets” OR “marketplace” OR “classifieds” OR “review sites” OR “cyber*”  OR “advertisements” OR “social networking sites” OR “labor markets”)  AND  (“prediction” OR “machine learning” OR “text mining” OR “natural language processing” OR “social network analysis” OR “data mining” OR “knowledge management” OR “deep multi modal models” OR “active learning” OR “sentiment analysis” OR “indicator mining” OR “statistical analysis” OR “network analysis”) |
| Note: The usage of shorter strings was done to fit in the search capability of the database namely Sciencedirect and Springerlink by ensuring the core concepts are retained and overlap the consistent concepts. | |

**Table C. Database search strategies and results**

| **Database** | **Search field** | **Filters** | **Query used** | **Raw records identified** | **Records retained after relevance-based pre- screening** |
| --- | --- | --- | --- | --- | --- |
| SpringerLink | Article, Conference Paper | English; 2002–2025 | Short query | 1488 | 55 |
| ScienceDirect | Research Articles | English; 2002–2025 | Short query | 773 | 47 |
| IEEE Xplore | All metadata | English; 2002–2025; Conferences & Journals | Long query | 8112 | 1008 |
| ACM Digital Library | Full-Text Collection, search within: full text | English; 2002–2025 | Long query | 442 | 309 |
| Taylor & Francis Online | Anywhere | English; 2002–2025 | Long query | 1235 | 1169 |
| Scopus | Document type: Article | English; Article; 2002–2025 | Long query | 3791 | 122 |

This table reports the raw returns from each database search, followed by the records retained after relevance-based pre-screening. Records were pre-screened at export by title-level topical relevance; records with no apparent relation to labor exploitation, trafficking, or computational detection methods were not retained. Following removal of 559 duplicates, 2,151 records proceeded to the screening stage shown in the PRISMA flow diagram.

**Query Example: Scopus query (database-specific syntax)**

( "labor trafficking" OR "modern slavery" OR "trafficking" OR "forced labor" OR "forced labour" OR "child labour" OR "trafficking networks" OR "human trafficking" OR "labour trafficking" OR "child labor" ) AND ( "dark web" OR "social media" OR "supply chain" OR "dark net" OR "virtual markets" OR "marketplace" OR "classifieds" OR "review sites" OR "cyber*" OR "advertisements" OR "social networking sites" OR "labor markets" ) AND ( "prediction" OR "machine learning" OR "text mining" OR "natural language processing" OR "social network analysis" OR "data mining" OR "knowledge management" OR "deep multi modal models" OR "active learning" OR "sentiment analysis" OR "indicator mining" OR "statistical analysis" OR "network analysis" ) AND PUBYEAR > 2001 AND PUBYEAR < 2026 AND ( LIMIT-TO ( DOCTYPE , "ar" ) ) AND ( LIMIT-TO ( LANGUAGE , "English" ) )

Search results: 3791
