# Supplementary material for: Online labor trafficking and machine learning: A scoping review and research agenda
Source: PLoS One. 2026 Sep 24;21(9):e0358425. doi: 10.1371/journal.pone.0358425 (PMC13606961; doi:10.1371/journal.pone.0358425)
Supplement: Supplementary file 3 [file pone.0358425.s002.docx]

**Supporting Information**

**S2 File. Review protocol summary**

**Eligibility, screening, charting and classification criteria**

This file summarises the protocol developed a priori by the author team, before the searches were run for conducting the review study.

Search strategy | Databases, search fields, filters, date range and full search strings are reported in S1 File.

| **Protocol element** | **Specification** |
| --- | --- |
| **Operational eligibility criteria** |  |
| Screening procedure | 1. Pre-screening at export by title-level topical relevance 2. Records with no apparent relation to exploitation, trafficking or computational analytics, not retained. 3. Retained records screened at title/abstract 4. Retained records screened at full text |
| Criterion A: applies or advocates ML or analytics | 1. Applies: implements and reports a computational model or analytic procedure on data. 2. Advocates: proposes a specific method together with a named data source and detection task. |
| Criterion B: methodological transferability | 1. The data source type has already been explored, or is plausibly explorable, for labor trafficking 2. the machine learning or analytics approach demonstrated in the adjacent context could be similarly designed for labor trafficking detection |
| Criterion C: minimum reporting | The source reports its data source, the method or model applied, and the basis on which results were evaluated. All three elements were required, regardless of source type. |
| Context criterion | Trafficking signals are derivable from online or online-accessible data, including recruitment boards, job reviews, web content, open geodata and remotely sensed data. Covers both IF-LT and OA-LT. |
| Exclusion criteria | Conceptual or theoretical treatments without any advocacy for machine learning or analytics; sex-trafficking studies without a transferable method; studies where trafficking signals are not derivable from online or online-accessible data; sources not in English. |
| **Source-type eligibility** |  |
| Eligible source types | 1. All source types must meet criterion A. 2. Grey literature in the included set comprises the preprints and dissertations. Additional grey literature, namely agency and institutional publications, supports definitional, contextual and indicator-framework claims and was not screened against these criteria. 3. Source types:  - Peer-reviewed journal articles (n = 9); - peer-reviewed conference papers, including workshop papers with a documented review process (n = 7); - preprints deposited in a recognised preprint server, included only where no peer-reviewed version was identified at screening and substituted where one was found (n = 3); - doctoral dissertations from an accredited institution, publicly retrievable in full text (n = 2); |
| Transferable studies from adjacent domains | - Peer-reviewed studies from adjacent exploitation contexts, primarily sex trafficking, n = 7 - Retrieved by the main search strings; retained following targeted exploration of their methodological relevance at full-text screening - Assessed against criterion B - Charted in Table 1 and flagged in the MT column; excluded from the labor trafficking count |
| **Data charting instrument** |  |
| Charted fields | 1. Author (year); title; research type; data source; country; techniques and methods; key findings. 2. Country was recorded as ‘Global’, with the scope described, where no single country applied. |
| LT (direct); MT (methodologically transferable) | 1. Dropdown: Yes; No. 2. The applicable designation is marked in Table 1. |
| **Classification rules** |  |
| LT versus MT | 1. A study was charted as LT where it addressed online labor trafficking directly, and as MT where it did not but met criterion B. 2. Illicit massage businesses were classified under LT rather than sex trafficking, following Polaris hotline findings in which survivors reported some form of labor trafficking in all cases. |
| IF-LT versus OA-LT | A study was thematically assigned at the synthesis stage to   1. IF-LT where recruitment or coordination occurs through digital platforms, and to 2. OA-LT where the operation occurs offline but signals can be retrieved online. |
